# HiCat: a semi-supervised approach for cell type annotation

**DOI:** 10.1093/bib/bbaf428

**Published:** 2025-08-20

**Authors:** Chang Bi, Kailun Bai, Xuekui Zhang

**Affiliations:** Department of Mathematics and Statistics, University of Victoria, 3800 Finnerty Road, Victoria, BC V8P 5C2, Canada; Department of Mathematics and Statistics, University of Victoria, 3800 Finnerty Road, Victoria, BC V8P 5C2, Canada; Department of Mathematics and Statistics, University of Victoria, 3800 Finnerty Road, Victoria, BC V8P 5C2, Canada

**Keywords:** cell annotation, semi-supervised learning, single-cell RNA sequencing, transformative embeddings

## Abstract

Existing cell type annotation methods face significant hurdles: supervised approaches often fail to differentiate between novel cell types not present in reference data, while unsupervised techniques can suffer from cluster impurity and difficulties in robustly distinguishing multiple distinct unknown cell populations. This critical gap motivated the development of HiCat, a semi-supervised pipeline specifically designed to overcome these limitations. HiCat is a semi-supervised pipeline that integrates both approaches, leveraging reference (labeled) and query (unlabeled) genomic data to simultaneously enhance annotation accuracy for known cell types and improve the discovery and differentiation of novel ones. HiCat follows a structured pipeline: (1) removing batch effects and generate a low-dimensional embedding; (2) nonlinear dimensionality reduction for capturing key patterns; (3) unsupervised clustering for proposing novel cell type candidates; (4) merging multi-resolution features from previous steps into a condensed feature space; (5) training a classifier on reference data for supervised annotation; and (6) resolving inconsistencies between supervised predictions and unsupervised clusters to finalize annotations, particularly for unseen types. Performance was evaluated across 10 public genomic datasets and perform a case study on a molecular cell atlas of the human lung. HiCat demonstrated superior performance in both known cell type classification and novel cell type identification. In benchmark evaluations, HiCat consistently outperformed existing methods, critically excelling in identifying and distinguishing multiple novel cell types. HiCat presents a robust framework for scRNA-seq cell annotation, improving classification accuracy and novel type identification. In addition, it provides a scalable and transferable solution for biomedical research, directly addressing key challenges in automated cell annotation.

## Introduction

Single-cell RNA sequencing (scRNA-seq) has revolutionized bioinformatics and biomedicine by providing unprecedented insights into cellular heterogeneity, rare cell types, and cell-to-cell interactions [1, 2]. This technology is pivotal for advancing our understanding of complex biological systems and diseases. In cancer research, e.g. scRNA-seq enables researchers to dissect tumor heterogeneity, offering a clearer picture of how diverse cell populations contribute to disease progression and treatment resistance [3, 4]. Its applications extend to developmental biology, where scRNA-seq helps elucidate the dynamic processes of cell differentiation, and immunology, by revealing the intricate landscape of immune cell diversity and function [2]. These insights are crucial for developing targeted therapies and personalized medicine approaches.

Unsupervised machine learning, particularly through clustering analysis, is a prominent method for annotating cell types. These techniques cluster cells using a gene-by-cell expression matrix and subsequently label each resulting cluster. Despite their effectiveness and popularity, these methods encounter two significant limitations. First, clustering annotations often depend on feature genes from the literature, making the process labor-intensive and heavily reliant on expert input. This dependency raises issues with scalability and reproducibility [5, 6]. Recently, anchor-based annotation approach has recently emerged as a partial solution, exemplified by Seurat version 3 or higher version [7], leveraging another single-cell RNA-seq dataset (known as the reference set) that includes annotated cell type labels. Cells in the reference set are grouped by their cell types, and each cluster is anchored to specific cell type groups in the reference set and labeled accordingly. Second, unsupervised annotation is often prone to errors from cluster impurity, leading to misannotations [5], an issue that lacks a satisfactory remedy. Such challenges can hinder the discovery of new cell types and complicate our understanding of biological processes. With the increasing amount of scRNA-seq data, there is a critical need for enhanced annotation methods that are both efficient and accurate [6].

With the growing use of single-cell sequencing technology, an increasing amount of annotated single-cell genomic data has become available to researchers. This trend has driven the development of supervised learning techniques for cell type annotation in single-cell genomics. These methods leverage labeled reference datasets to classify individual cells in query data, thereby addressing issues of cluster impurity by annotating cells rather than entire clusters. Various general-purpose supervised machine learning models have been employed to create cell annotators. For instance, Garnett [8] use Elastic Net for cell type classification. CaSTLe [9] uses XGBoost [10], and SingleCellNet [11] applies random forests [12] for cell classification based on selected genes and derived features. scPred [13] and Moana [14] utilize support vector machines [15] for classification. Correlation-based approaches have also been widely used for cell annotation, as seen in methods such as SingleR [16], scmap-cluster [17], CHETAH [18], scMatch [19], ClustifyR [20], and CIPR [21]. K-nearest neighbor classifiers [22] form part of the pipelines in scmap-cell [17], Moana [14], OnClass [23], scANVI [24], and scClassify [25]. scID [26] employs linear discriminant analysis for cell type annotation, while scAnnotate [27] incorporates generative learning and ensemble techniques. To mitigate batch effects between reference and query datasets, scMagic [28] performs a second round of reference-based classification. In addition to traditional machine learning and statistical methods, deep learning approaches have also been developed for cell annotation, including SuperCT [29], ACTINN [30], scDeepSort [31], and EpiAnno [32].

Supervised cell annotation methods classify cells based on known cell types present in the reference set, making them unable to identify truly novel cell types. Some methods, such as Scmap [17], CHETAH [18], SingleCellNet [11], scClassify [25], and scID [26], can assign an “unassigned” label when a cell is considered too dissimilar to any known types. While this feature can help in detecting novel cell types, it is not ideal because these methods optimize feature selection primarily for distinguishing between known categories. In real data experiments, we observed a decline in annotation performance as the proportion of unknown cell types increased. Additionally, these methods are unable to differentiate between multiple distinct unseen cell types, a capability that is a strength of unsupervised approaches. This suggests that using semi-supervised learning methods, which combine the benefits of both supervised and unsupervised approaches, may provide a more effective solution.

Semi-supervised learning combines the strengths of supervised and unsupervised learning to improve cell type annotation accuracy. It leverages labeled reference data while incorporating information from unlabeled data, resulting in more robust predictions even when labeled data are limited. This is particularly valuable in scRNA-seq contexts where cell type diversity is vast, and labeled data availability is limited [6]. Recently semi-supervised methods were developed for cell annotation. For example, CALLR [33] utilizes unlabeled data by combining a sparse logistic regression and a Laplacian matrix constructed from all cells for clustering. scNym [34] adapts pseudo-labeling technique and Domain Adversarial Network [35] to incorporate unlabeled data into the training process. scSemiGan [36] uses a modified Generative Adversarial Network [37] that uses labeled data to guide the training process, while leverages unlabeled data by generating latent representations that capture underlying structures. scBERT [38] performs a self-supervised pre-training on unlabeled data then fine-tuning the model on labeled data.

While semi-supervised learning offers a promising direction, a critical gap remains: existing methods largely focus on improving the classification of known cell types and do not adequately address the challenge of systematically identifying, and crucially, differentiating between multiple distinct novel cell types. Many do not explicitly implement functionalities for such fine-grained novel discovery. Furthermore, most current approaches operate on feature spaces of a single resolution, potentially overlooking the benefits of integrating information from features at multiple resolutions, which can capture different facets of cellular identity.

The novelty of HiCat lies in its integrated semi-supervised pipeline specifically architected to address these deficiencies. HiCat’s distinct contributions include the following:

- **A structured, sequential approach to feature engineering that creates a unique multi-resolution feature space.** This space combines batch-corrected principal components (PCs), Uniform Manifold Approximation and Projection (UMAP) embeddings, and unsupervised cluster identities, providing a rich substrate for the classifier.
- **The synergistic use of a CatBoost classifier with this multi-resolution input**, allowing for automated selection of the most relevant features at different resolutions for distinguishing cell types.
- **A novel decision-making process that intelligently fuses supervised predictions with unsupervised clustering information.** This not only ensures high accuracy for known cell types but, critically, enables the identification and distinct labeling of multiple novel cell populations based on confidence scores and unsupervised cluster assignments.
- **The explicit capability to distinguish between multiple, different unseen cell types within the query data,** a feature largely unaddressed by previous methods.

## Materials and methods

The input data of cell annotation consist of two genomic matrices: one from the reference set and the other from the query dataset. In these matrices, the rows represent tens of thousands of genes, while the columns indicate cells, typically numbering in the hundreds of thousands. The input also includes cell type labels for each cell in the reference set, but the query data do not have these labels. The main goal of the cell annotation analysis is to predict these absent labels.

**Figure 1:**
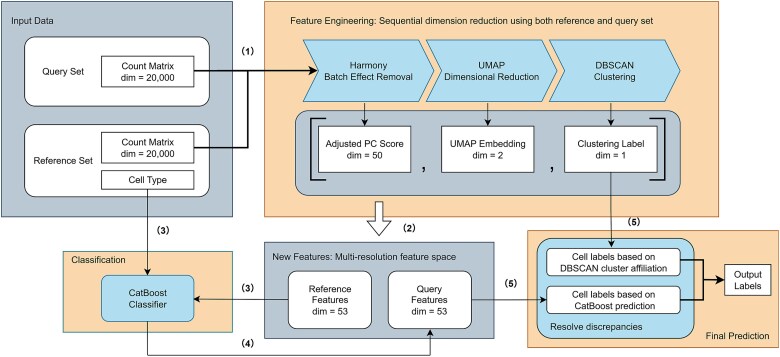
Workflow of HiCat: **(1) Sequential dimension reduction:** Harmony is applied to remove batch effects between the genomic matrices of the reference and query sets, reducing the data to 50 PCs. The data are then further reduced by UMAP and clustered using DBSCAN. **(2) Concatenation of multi-resolution features:** the aligned PC scores, UMAP embeddings, and cluster labels are combined to create a 53D multi-resolution feature space derived from both reference and query datasets. **(3) Model training:** the 53D features along with the cell type labels from the reference set are used to train a CatBoost classifier. **(4) Classification of query cells:** the trained classifier is applied to the new features of the query set, providing cell type annotations with predictive probabilities as indicators of confidence. **(5) Resolving discrepancies:** discrepancies between the DBSCAN and CatBoost outputs are resolved to determine the final cell type labels; the CatBoost prediction is used when its predictive probability is high, otherwise, the DBSCAN label is assigned.

HiCat (Hybrid Cell Annotation using Transformative embeddings) is a semi-supervised framework created for annotating cell types in scRNA-seq data. Figure 1 illustrates the different components of the HiCat pipeline. In the rest of Method section, we present a comprehensive overview of these components in six analysis steps, concluding with a summary of the method’s innovative aspects.

Following the identification of common genes between the reference and query datasets, and data normalization, highly variable genes (HVGs) were selected from the combined data using Seurat’s FindVariableFeatures function. The subsequent principal component analysis (PCA), which is the initial step for Harmony, was performed on these HVGs. The top 50 PCs derived from these HVGs were then used as input for the Harmony algorithm to correct for batch effects.

**Step 1. Batch effect removal using Harmony** To align the reference and query datasets, we start by using Harmony [39], an algorithm developed to correct batch effects in multi-dataset integrations. We choose Harmony since it was recommended as top 3 batch effect removal methods in a recent benchmark study [40] Harmony functions within the PCs space, iteratively adjusting the data to synchronize shared cell types across datasets, all while preserving biological variation. In our pipeline, we apply Harmony on the top 50 PCs, its default configuration, for both the reference and query datasets. This ensures that the shared patterns are retained as batch-specific variation is eliminated. The outcome is a harmonized 50D embedding for both datasets. We input the reference and query datasets containing tens of thousands of genes, and the result is a 50D PC embedding that forms the basis for further analysis.

**Step 2. Dimensionality reduction using UMAP** Following Harmony-based alignment, we further reduce the data’s dimensionality using UMAP [41]. We opt for UMAP, the leading method for reducing dimensions in visualizing single-cell genomic data. It is a nonlinear technique for dimensionality reduction that effectively captures both local and global structures within high-dimensional datasets. We apply UMAP to the 50D embedding obtained in the prior step, compressing it down to two dimensions to reveal the two most critical patterns in the data. The input for this process is the 50D embedding produced by Harmony, while the output is a 2D UMAP embedding that highlights the most critical data patterns.

**Step 3. Clustering with DBSCAN** Next, we conduct unsupervised clustering on the 2D UMAP embeddings using Density-Based Spatial Clustering of Applications with Noise (DBSCAN) [42]. DBSCAN was chosen over other common clustering algorithms (e.g. K-means, graph-based methods like Leiden/Louvain, or Gaussian Mixture Models) for several key advantages pertinent to HiCat’s goal of identifying potentially unknown and rare novel cell types.

Unlike methods requiring a predefined number of clusters (e.g. K-means, often GMMs) or those that partition all cells into groups (e.g. Leiden/Louvain), DBSCAN can identify clusters of varying shapes and densities without prior specification of their quantity. It excels at distinguishing noise or outlier cells and can leave some cells unassigned, which is valuable for highlighting ambiguous cases or rare populations that might be obscured by methods forcing all cells into clusters. While graph-based methods are powerful for community detection, and GMMs for model-based clustering, DBSCAN’s parameters (epsilon distance and minimum samples) offer a more direct way to tune sensitivity for detecting smaller, dense, and potentially novel cell groups that might not conform to global graph structures or Gaussian assumptions. These characteristics make DBSCAN particularly well-suited for HiCat’s objective of *de novo* pattern discovery in the UMAP space, complementing the supervised learning components. The biological interpretation and relevance of clusters identified by DBSCAN are further assessed within HiCat through integration with supervised predictions (Step 6) and validated against ground-truth annotations in our benchmarks.

DBSCAN produces a 1D vector where each cell is assigned a cluster label or marked as noise if it does not fit into any cluster. This vector serves as one of the features in the subsequent multi-resolution feature space.

**Step 4. Feature space concatenation** To fully integrate biological and structural insights from both datasets, we combine the results from steps 1 to 3. This process merges the 50D PC embedding from Harmony, the 2D UMAP embedding, and the 1D cluster membership vector from DBSCAN. The result is a 53D feature space representing various resolution levels, encompassing known and unknown patterns from the reference and query datasets. The inputs for this phase include the outputs from Harmony, UMAP, and DBSCAN, which together form this 53D feature space for the genomic data of both reference and query datasets. This multi-resolution feature space is designed to provide the classifier with a rich, condensed representation of cellular identity. This approach moves beyond the high-dimensionality and inherent noise of raw gene expression data, aiming to capture more stable and informative biological signals at various resolutions.

**Step 5. Supervised learning using CatBoost** For supervised cell types annotation, we train a CatBoost classifier [43] with the reference dataset and its corresponding cell type labels. CatBoost is a gradient boosting algorithm that performs exceptionally well with categorical features and includes built-in regularization to prevent overfitting. Using the 53D feature vectors from the reference dataset, the model is trained to predict cell type labels using the extracted features. Once trained, the model is used to predict the cell type for each cell in the query dataset, along with providing a probability distribution across potential cell types. For this step, the inputs are the 53D feature vectors and reference cell type labels, while the output is a trained CatBoost model and a probability distribution of cell types for each cell in the query set.

**Step 6. Resolving discrepancies between supervised and clustering-based labels** In Step 3, each cell is annotated based on its cluster affiliation, while in Step 5, cell types are assigned based on the highest predicted probability from CatBoost. CatBoost and DBSCAN work integrally to reinforce each other. The cluster affiliations serve as a strong guide for the classifier, improving the accuracy when predicting known cell types; the classifier discriminates low confidence prediction, allowing the cluster affiliation to only apply to cells with potentially unseen types in the final prediction. The predicted probability from CatBoost reflects its confidence in classification. If this probability is close to “1/number of cell types,” it indicates low confidence in CatBoost’s prediction, and we defer to DBSCAN’s cluster affiliation. Conversely, if the predicted probability is close to 1, it signifies high confidence in CatBoost’s result, which should be used. The threshold for determining high confidence is based on the largest drop in predicted probabilities across both the reference and query datasets. The input to this step includes the CatBoost predictions and the DBSCAN cluster labels, and the final output is the annotated cell type labels for the query dataset.

This step is crucial for interpreting the biological significance of DBSCAN clusters, ensuring that novel type assignments are made only when supervised evidence for known types is weak, and allows for their subsequent validation.

## Results

We utilize 10 published single-cell genomic datasets that often serve as benchmarks for other cell annotation methods. These datasets represent a range of mouse and human tissues and were sequenced via different protocols. They consist of datasets from the mouse pancreas [44], the human pancreas [44–49], Tabula Muris (TM) [50], PBMC [51], and the mouse brain [52, 53]. From these datasets, we derive 33 independent pairs of reference and query sets for external validation. These pairs are structured to ensure the two sets are comparable, e.g. both from the pancreas or both from PBMC, but not a pancreas reference paired with a PBMC query. Supplementary table, datasetDetails.pdf, outlines the detailed information of these 33 pairs from the datasets used in our first two experiments. The cell type labels of the query sets serve as the ground truth for assessing the classification performance of the predicted labels from the annotation methods. Further details on dataset acquisition and preprocessing can be found in Bai *et al*. [54], as this benchmark closely follows the procedures outlined in that paper.

To evaluate HiCat’s performance, we compare it with well-established methods, including Seurat [55], SingleCellNet [11], scClassify [25], SingleR [16], CaSTLe [9], SCINA [56], scID [26], CHETAH [18], and scmap [17]. Our assessment consists of three experiments: (1) measuring the annotation performance of all methods when the query data have no unseen cell types. (2) Analyzing their annotation performance with query sets that contain unseen cell types; here, SingleCellNet [11], scClassify [25], scID [26], SCINA [56], and CHETAH [18] are evaluated alongside HiCat, due to their ability to assign “unassigned” or “unknown” labels to samples when query samples are dissimilar to any known types in reference set in their default setting. (3) Evaluating performance in distinguishing multiple unknown cell types. Notably, the mentioned methods do not distinguish between cell types in unknown classes, highlighting a key feature of HiCat. Consequently, we will specifically showcase HiCat’s outcomes in this experiment. All methods are used with their default settings to prevent bias from our ability to fine-tune different methods.

### Performance of annotating seen cell types

**Figure 2:**
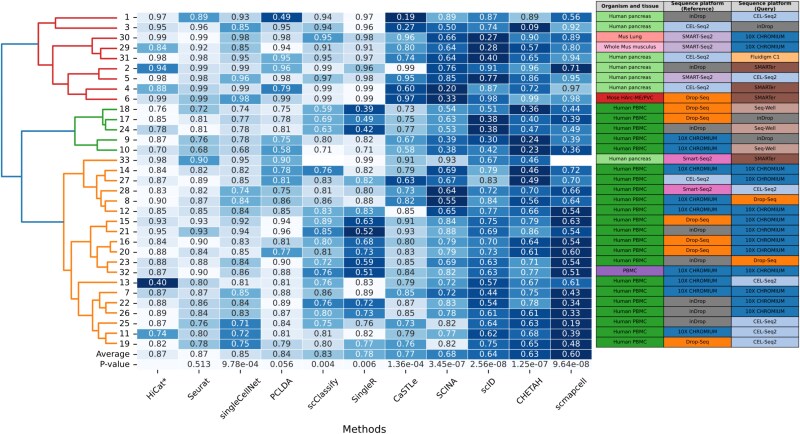
Performance evaluation on data without unseen cell types. Each row depicts a pair of reference and query sets. The columns illustrate the performance of different methods, with each cell’s number reflecting the accuracy of the method applied to the corresponding dataset pair. The last row displays the average accuracy for all datasets per method. The color coding indicates the ranking of methods within each dataset, where the lighter hues signify superior performance. The findings indicate that HiCat consistently ranks the highest across the majority of datasets. The $P$-value represents the paired Wilcoxon signed-rank test against HiCat for each method. A hierarchical clustering is performed in row to group similar performing dataset pairs together. We notice that, in general, the performance clusters are primarily correlated to the organism and tissue of the data pairs. While some methods showed to be optimized toward certain data conditions, HiCat’s performance remains similar across all dataset pairs. This indicates that HiCat generalized well in variety of conditions.

The first experiment assesses how effectively HiCat annotates cells in the query set that match cell types found in the reference set. In each dataset pair, we designate the dataset with a greater variety of cell types as the reference and the other as the query set, ensuring that the query set does not include any unseen cell types. We use the overall accuracy as the evaluation metric for this task, which is defined as the proportion of cells assigned with the correct cell type. As illustrated in the heatmap in Fig. 2, HiCat demonstrates highly competitive performance, consistently ranking among the top methods across the 33 dataset pairs. While HiCat achieves the highest average accuracy overall, a paired Wilcoxon signed-rank test shows its performance is statistically on par with other leading methods like Seurat for this specific task. This result is important because it establishes that HiCat’s complex, semi-supervised framework does not compromise its ability to perform standard classification. It functions as a state-of-the-art classifier for known cell types, providing a reliable foundation before considering its more advanced novel discovery capabilities.

### Performance of annotation when unseen cell types present

In our second experiment, we assessed HiCat’s annotation performance when a query dataset contained cell types not present in the reference. To do this, we established three scenarios by randomly holding out one, two, or three cell types from the reference dataset, respectively. Our evaluation focuses on two main aspects of annotation performance. First, we measure the overall accuracy when unseen types are present, calculated similarly to the first experiment but with an additional category labeled “unseen” or “unassigned,” representing cells that do not match any cell type in the reference dataset. Second, we evaluate the ability to identify cells as “unseen,” treating this as a binary classification problem and utilizing the F1 score, the harmonic mean of precision and recall, a common metric in machine learning for binary classification. Among the methods compared with HiCat in the first experiment, six of them (scmapcell, CHETAH, SCINA, scClassify, singleCellNet, scID) can assign an “unassigned” label for cells that cannot be confidently annotated, which we interpret here as an “unseen” label. HiCat specifically labels cells as “unseen” and, when appropriate, categorizes them into subgroups if multiple unseen cell types are detected. However, for the purpose of this analysis, we simplify by treating all unseen cell types as a single category. During the evaluation on the query set, all cell types that are not present in the reference set are mapped to the single “unseen types”, and the overall accuracy and F1 score are then calculated as in supervised learning way.

**Figure 3:**
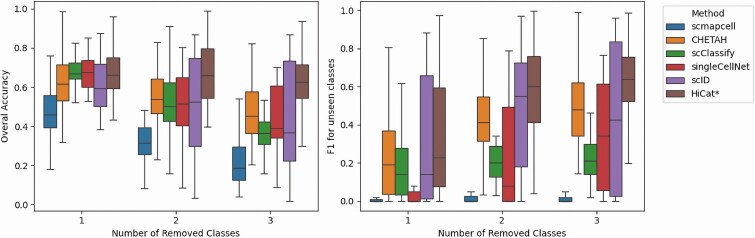
Annotation performance on data with unseen cell types. Each boxplot displays the performance of the respective method across all 33 dataset pairs. The horizontal axis represents the number of unseen cell types in the query set, created by randomly omitting cell types from data. The left panel shows overall accuracy, reflecting each model’s ability to classify all cell types, categorizing missing reference types as “unseen”. The right panel shows the F1-score for the “unseen” category, based on precision and recall in identifying these cells. Results indicate that HiCat consistently outperforms other methods when unseen cell types exist.

The left panel of Fig. 3 illustrates the overall accuracy of HiCat in comparison with six alternative methods. Each boxplot summarizing the accuracy of a method across 33 data pairs. All six methods demonstrate lower accuracy than in the first experiment, suggesting that the presence of unseen cell types hampers annotation performance universally. HiCat clearly excels, securing the highest overall accuracy. Interestingly, HiCat’s median accuracy remains largely stable despite the increase in unseen cell types, while the accuracy of other methods tends to decline as more unseen cell types are introduced. This implies that the other methods struggle, leading to a higher frequency of incorrect annotations for unseen types, whereas HiCat’s robust performance in identifying new cells allows it to sustain relatively steady results. This resilience to out-of-distribution data is a key enhancement offered by HiCat’s semi-supervised framework, demonstrating its reliability in realistic discovery scenarios.

The right panel of Fig. 3 presents the F1 scores for each method’s binary classification in identifying unseen cells, with HiCat once again being the leading performer. The relationship between the number of unseen cell types and the F1 score differs from the trend observed for overall accuracy, as the F1 score represents a balanced summary of precision and recall and is influenced by the class distribution. In this experiment, the proportion of unseen cells is relatively small compared with the total number of cells. As more cell types are removed, the balance between seen and unseen cells improves, impacting the F1 scores. Regardless of these dynamics, HiCat’s leading F1 score underscores its superior ability to correctly identify novel cells with high precision and recall, a critical benefit for any discovery-focused analysis.

### Distinguishing multiple unseen cell types

In the third experiment, we demonstrate HiCat’s ability to distinguish between multiple unseen cell types, a significant advancement over existing methods that can only group novel cells into a single “unknown” category. As this capability is unique to HiCat, no comparison is made with other methods. We illustrate this using examples from two data pairs. The pancreas dataset from Xin *et al*. [46] is selected as the reference set because it includes cell types (alpha, beta, delta, and gamma) commonly found in other pancreas datasets. Two pancreas datasets, Muraro *et al*. [45] and Segerstolpe *et al*. [48], are used as query sets because they contain all cell types present in the Xin *et al*. [46] dataset as well as several additional cell types, such as acinar, ductal, stellate, and endothelial.

**Figure 4:**
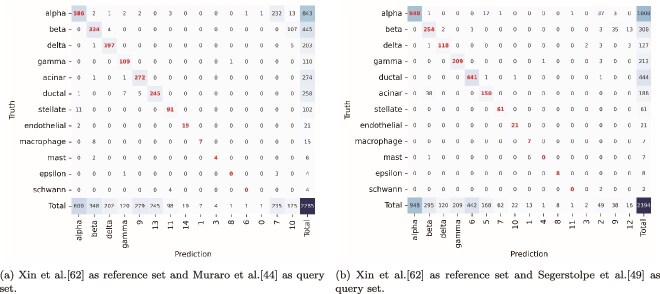
Confusion matrices for cell annotation involving multiple unknown cell types are shown. The rows correspond to the cell labels from the query set, based on published data considered as the ground truth, while the columns represent the cell types predicted by HiCat. Since the reference set contains only four cell types, any additional, unseen types are assigned numerical labels as cell type IDs in the columns. Each element in the confusion matrix indicates the number of cells in the query set with a published label (row) that matches a predicted label (column). The left panel (a) presents the results using Muraro *et al*. [45] as the query set, while the right panel (b) shows the results using Segerstolpe *et al*. [48] as the query set. Cluster labels are arranged to align as closely as possible with the likely true labels, with the counts concentrated along the diagonal indicating correct annotations. Leveraging the strength of DBSCAN in detecting small clusters, HiCat successfully distinguished many unknown cell types, even when they contained fewer than 10 cells.

Figure 4 presents the confusion matrices for cell annotation on two query datasets. Row names are the labels provided by published data, which are used as ground truth. Column names are HiCat’s predicted labels. The numbers along the diagonal (highlighted by the red text) represent the count of correctly annotated cells, while the off-diagonal numbers indicate misannotated cells. In this analysis, we focus on HiCat’s performance in distinguishing unseen cell types, which are assigned numerical ID labels for predicted cell types. In both query sets, there are four larger unseen cell types, ranging from 21 to 444 cells, including acinar, ductal, stellate, and endothelial. HiCat accurately annotated all of these cell types, achieving over 90% accuracy and assigning distinct numerical IDs to each type. For instance, in Fig. 4(b), the ductal cells were the best-annotated, with 441 out of 444 cells correctly identified as cluster 6, corresponding to 99.3% sensitivity. Moreover, only one cell within cluster 6 was not ductal, resulting in a high specificity of 99.8%. It is impressive to show HiCat’s ability to consistently identify the unseen cell type (endothelial) with high accuracy in both query sets, despite having only about 20 cells in both query sets. This high concordance between HiCat-identified novel clusters (which are initially delineated by DBSCAN and refined through our discrepancy resolution step) and the expert-annotated ground-truth cell types in the query datasets serves as a strong validation of the biological relevance of the clusters identified by our pipeline. This ability to provide a granular, multi-class annotation of novel populations is a key benefit of HiCat, offering researchers a much more detailed and actionable view of cellular diversity in their data.

### HiCat’s errors and potential solutions for improvement

We observed HiCat’s three types of errors from Fig. 4. We discuss these observed errors and suggest potential approaches for addressing them.

The first type of error involves unknown clusters (seen as columns on the right-hand side of each confusion matrix) that should belong to a known cell type, such as alpha or beta. This issue arises from the heterogeneity within these cell types, where certain subtypes exhibit gene expression profiles that differ significantly from those observed in the reference data. These errors can often be resolved through manual annotation, using feature genes identified in the literature to recognize them as subtypes of known cell types, and subsequently merging them back into the correct categories. Although this type of error largely reduced HiCat’s accuracy in our evaluation, it can actually be seen as an advantage, as it allows for the identification of subtypes rather than being merely considered an annotation mistake. This capability can provide valuable insights into the underlying heterogeneity of cell populations.

The second type of error occurs when HiCat fails to separate similar cell types. For instance, macrophages and mast cells are correctly annotated without error in Muraro *et al*. [45] query set, but they are grouped into a single cluster in Segerstolpe’s query set. This reflects the known similarities in gene expression between macrophages and mast cells, which share roles in immune responses, inflammation, and the release of common cytokines, chemokines, and other mediators [57, 58]. To address this type of error, it is important to identify cell types with highly similar gene expression profiles and conduct downstream analyses to distinguish them further using curated marker genes specific to those cell types.

The third type of error involves identifying noise clusters containing only one or two cells, observed in both query sets. These clusters typically represent outliers from known cell types. While HiCat’s sensitivity to rare and unknown cell types enables it to detect novel patterns, it also makes it prone to recognizing outliers as distinct clusters. Consequently, caution is advised when interpreting clusters with very few cells, as they are more likely to reflect noise rather than meaningful biological variation.

### Impact of multi-resolution feature space on model performance

**Figure 5:**
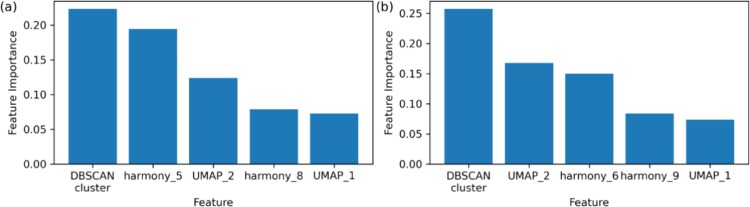
Top five features with the highest importance in CatBoost for Experiment 3 (a) and (b) correspondingly, accounting for 70% of total feature importance. These features span all three levels of resolution in both cases, with no single level dominating, indicating the classifier’s effective utilization of diverse the resolutions of the features.

The construction of a multi-resolution feature space is a unique and innovative aspect of HiCat. Its supervised learning component, CatBoost, uses numerous shallow trees (parsimonious models) that sequentially address misclassifications from prior ensemble models. Each tree is expected to automatically select important features from the multi-resolution feature space tailored to specific misclassifications. Figure 5 shows the top five features with the highest importance in two data analyses from experiment 3, demonstrating that features across all resolutions are selected by many shallow trees and play an essential role in HiCat’s predictions.

### Case study: novel cell type discovery in a large-scale human lung dataset

To further assess HiCat’s performance on a large and complex human dataset, and to address its utility in a realistic discovery scenario, we conducted a case study using a public human lung dataset (GEO: GSE135893) from a comprehensive lung cell atlas [59] collected from DISCO [60]. This dataset comprises 31,305 cells and 44 annotated cell types. We simulated a common research scenario where a reference atlas contains only the most abundant and well-characterized cell populations. To do this, we constructed a reference set for HiCat using only the 10 most abundant cell populations (including Alveolar macrophage, M1 macrophage, CD14 monocyte, Memory CD4 T cell, GZMB CD8 T cell, CD16 NK cell, AT2, Multiciliated cell, Capillary EC, Aerocyte). The entire dataset was then used as the query set, tasking HiCat with annotating the known reference cells while discovering the remaining 34 cell types as “novel.”

The results demonstrated HiCat’s ability to successfully identify novel cell populations with varying degrees of granularity, reflecting known biological principles and computational challenges in scRNA-seq analysis. First, HiCat successfully identified Mast cells, a transcriptionally distinct novel population, and correctly assigned them to a unique cluster (cluster 10). This demonstrates the pipeline’s core capability to discover and delineate a novel cell type when its expression profile is clearly distinct from the provided reference set.

Furthermore, the experiment highlighted HiCat’s ability to identify meaningful higher-order groupings of related novel cell types that share strong biological signatures. For instance, Cycling T/NK cells and G2/M phase myeloid cells were grouped into a single novel cluster (cluster 2). This outcome is consistent with known analysis challenges, as the powerful, shared gene expression signature of cell proliferation is a dominant source of variation that can mask underlying lineage-specific signals [61]. Similarly, multiple fibroblast subtypes, pericytes, and vascular smooth muscle cells were grouped into a single “stromal” cluster (cluster 5), which aligns with their shared mesenchymal lineage described in the lung cell atlas [59]. Various B cell maturation states were also grouped into a “B-cell lineage” cluster (cluster 7), reflecting their single developmental continuum [62]. This indicates that HiCat effectively captures broad biological themes and cell lineage relationships among the unknown cell populations.

This case study also characterized important performance boundaries of the HiCat framework. We observed that novel cell subtypes were often “collapsed” into their parent lineage if that parent type was present in the reference set. For example, AT1 cells were classified as the known AT2 type, reflecting their shared alveolar epithelial lineage; Arterial EC and Venous EC were classified as the known Capillary EC, their parent endothelial type; and Treg cells were classified as the known Memory CD4 T cells, consistent with their shared T-cell identity [59]. This suggests that when a novel subtype is transcriptionally very similar to an abundant reference population, HiCat’s supervised component may confidently assign the parent label, overriding the novel discovery process [63]. This defines a practical limit where HiCat’s discovery power is strongest for lineages clearly distinct from the reference. Finally, predictable outcomes were observed for extremely rare populations ($n\! <\! 20$), which were sometimes grouped into small, heterogeneous clusters or absorbed into larger neighboring clusters. This reflects a known challenge in clustering sparse data and highlights a practical lower limit on cell count required for robust *de novo* identification [64].

**Figure 6:**
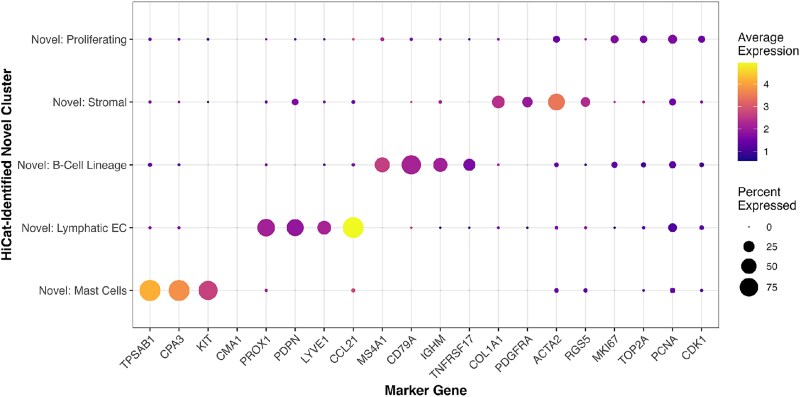
Biological validation of HiCat-identified novel clusters using canonical marker genes. The dot plot shows marker gene expression for the novel clusters identified in the human lung dataset case study. The dot size corresponds to the percentage of cells in each cluster expressing the gene, while color intensity reflects the average expression level. The specific expression patterns confirm the biological identity of each discovered population, such as the high expression of mast cell markers (e.g. TPSAB1, CPA3) exclusively in the “Novel: Mast Cells” cluster.

A detailed summary of HiCat’s performance on each withheld cell type is provided in Table 1. This case study validates HiCat’s utility on a large-scale dataset, showing that it successfully discovers biologically coherent cell groupings. To provide biological validation for these findings, we visualized the expression of canonical marker genes, selected based on established literature and the original lung atlas publication [59, 65–68], for the major novel clusters HiCat identified (Fig. 6). The dot plot confirms that each novel cluster specifically expresses markers for its corresponding identity—for instance, the “Novel: Mast Cells” cluster shows high specific expression of TPSAB1 and CPA3.

**Table 1:** Performance of HiCat in identifying withheld novel cell types in the human lung dataset case study

**Withheld cell type**	**HiCat’s assigned label**	**Cells mapped**	**Total cells**	**Recall** ^*^	**Precision** ^*^	**F1-score** ^*^
Mast cell	Novel Cluster 10	252	255	0.988	0.988	0.988
Lymphatic EC	Novel Cluster 8	456	458	0.996	0.983	0.989
*B-Cell Lineage*						
Memory B cell	Novel Cluster 7	205	225	0.911	0.507	0.652
Naive B cell	Novel Cluster 7	25	33	0.758	0.062	0.114
Plasma cell	Novel Cluster 7	165	174	0.948	0.408	0.571
*Stromal/Mesenchymal*						
Vascular smooth muscle cell	Novel Cluster 5	227	231	0.983	0.316	0.478
CFD+MGP+ fibroblast	Novel Cluster 5	289	374	0.773	0.402	0.529
Pericyte	Novel Cluster 5	136	137	0.993	0.189	0.318
Myofibroblast	Novel Cluster 5	35	36	0.972	0.049	0.093
GPC3+ fibroblast	Novel Cluster 5	12	12	1.000	0.017	0.033
ADAMDEC1+ADAM28+ fibroblast	Novel Cluster 5	4	5	0.800	0.006	0.011
S phase GPC3+ fibroblast	Novel Cluster 5	2	4	0.500	0.003	0.006
*Proliferating Cells*						
Cycling T/NK cell	Novel Cluster 2	102	261	0.391	0.103	0.163
G2/M phase myeloid cell	Novel Cluster 2	41	60	0.683	0.041	0.078
*Rare/Low-Abundance Groupings*						
cDC1	Novel Cluster 0	7	12	0.583	0.004	0.007
Red blood cell	Novel Cluster 0	3	12	0.250	0.002	0.003
Neuron	Novel Cluster 8	6	14	0.429	0.013	0.025
*Withheld Types Collapsed into Known Reference Types*						
*Collapsed into Epithelial Types:*						
AT1	AT2	736	737	-	-	-
Goblet cell	AT2	322	566	-	-	-
Club cell	AT2	90	91	-	-	-
Airway basal cell	AT2	65	68	-	-	-
Cycling AT2	AT2	42	43	-	-	-
CDC20B+ Multiciliated cell	Multiciliated cell	20	27	-	-	-
*Collapsed into Endothelial Type:*						
Arterial EC	Capillary EC	795	824	-	-	-
Venous EC	Capillary EC	672	745	-	-	-
*Collapsed into Immune Types:*						
Treg cell	Memory CD4 T cell	148	171	-	-	-
Naive CD4 T cell	Memory CD4 T cell	488	521	-	-	-
GZMK CD8 T cell	GZMB CD8 T cell	171	369	-	-	-
LYVE1 macrophage	M1 macrophage	526	653	-	-	-
cDC2	M1 macrophage	509	675	-	-	-
mregDC	M1 macrophage	11	20	-	-	-
S phase myeloid cell	Alveolar macrophage	22	36	-	-	-
CD16 monocyte	CD14 monocyte	591	611	-	-	-

^*^Recall, Precision, and F1-score are calculated only for withheld types that were assigned to a novel cluster ID (e.g. “Novel Cluster 10”). These metrics are not applicable (-) when a withheld type was classified as one of the known cell types from the reference set.

## Discussion

We benchmarked our method against other leading approaches using 10 publicly available genomic datasets. The studies yielded three key findings: (1) among methods tested on query data without new cell types, all performed well, but HiCat consistently delivered superior accuracy and cell type separation in most data pairs. (2) When query sets included unknown cell types, the performance of all methods dropped; however, HiCat remained the most resilient, achieving notably higher accuracy than its competitors. (3) To our knowledge, other methods do not directly tackle the challenge of distinguishing multiple unknown cell types. Conversely, HiCat excelled in these situations, effectively identifying and annotating unseen cell types, even with a limited number of cells in the query data. This exceptional performance stems from three design advantages of our method, which are discussed below.

### Integration of reference and query information

A key novel aspect of HiCat’s design is its integration of genomic data from both reference and query sets, creating a unified embedding space. This hybrid approach offers two distinct benefits. First, by incorporating query samples into the feature engineering process, the effective sample size is significantly increased, allowing the model to learn from a richer set of patterns. Second, training the model on a unified embedding space makes it more transferable, enabling it to better classify samples in the query set. This combination of leveraging both reference and query data empowers HiCat to excel in scenarios where other methods struggle to identify meaningful patterns, especially when the query data includes new cell types.

### Sequential dimensionality reduction steps and multi-resolution feature space

Another significant advantage of our approach is the deliberate construction of a multi-resolution feature space through sequential dimensionality reduction, moving from high-dimensional gene expression to a more condensed and informative 53D representation. This process begins with selecting HVGs from the normalized, combined reference and query data, upon which PCA is performed as the first step for Harmony. This strategy offers several benefits over directly using conventional gene expression data for classification. Raw gene expression is often characterized by high dimensionality, sparsity, and considerable technical noise. Our sequential application of PCA (within Harmony), UMAP, and DBSCAN clustering concentrates salient biological information while mitigating these issues. The resulting 50D batch-corrected PCs capture broad transcriptional programs, the 2D UMAP embeddings reveal finer local and global manifold structures, and the 1D DBSCAN cluster label provides an interpretable unsupervised grouping related to cell states or types.

This 53D feature space, encompassing outputs from these distinct resolution levels, allows HiCat to effectively capture diverse facets of cellular identity. While HiCat does not directly use pre-defined marker gene lists as input features for its final classification model, this multi-resolution space implicitly captures their influence. Strong signals from co-expressed marker genes or cell identity gene programs inherently shape the PCs and the UMAP manifold structure. The CatBoost classifier, an ensemble of shallow decision trees, then automatically selects the most pertinent features from these 53 candidates at their varying resolutions for each classification task. This multi-resolution strategy enables HiCat to identify subtle patterns that models relying on single-resolution features (e.g. only genes or only high-dimensional PCs) might overlook, particularly in complex cell types or mixed populations.

Furthermore, this approach complements traditional marker gene-based annotation. HiCat first provides an objective, data-driven framework for delineating cell groups, including the crucial identification of novel cell types. Once these populations are defined by HiCat, standard differential gene expression analysis can be subsequently applied to identify their characteristic marker genes, thereby aiding in their biological interpretation and validation. This is especially powerful for newly discovered cell types where marker genes are not yet established. By learning on these integrated and transformed features, HiCat aims to build models that are more robust and generalizable across diverse single-cell datasets.

### Fusing supervised and unsupervised signals

The supervised CatBoost model is trained with labeled reference data, ensuring accurate identification of known cell types, while unsupervised clustering using DBSCAN effectively identifies unknown cell types. This combined embedding approach creates a more robust feature space that can manage situations with new or sparse cell populations. By integrating these signals, HiCat circumvents the typical drawbacks of solely clustering-based methods, which often face issues with cluster impurity, and purely supervised methods, which struggle to incorporate novel cell types.

### Computational considerations

**Figure 7:**
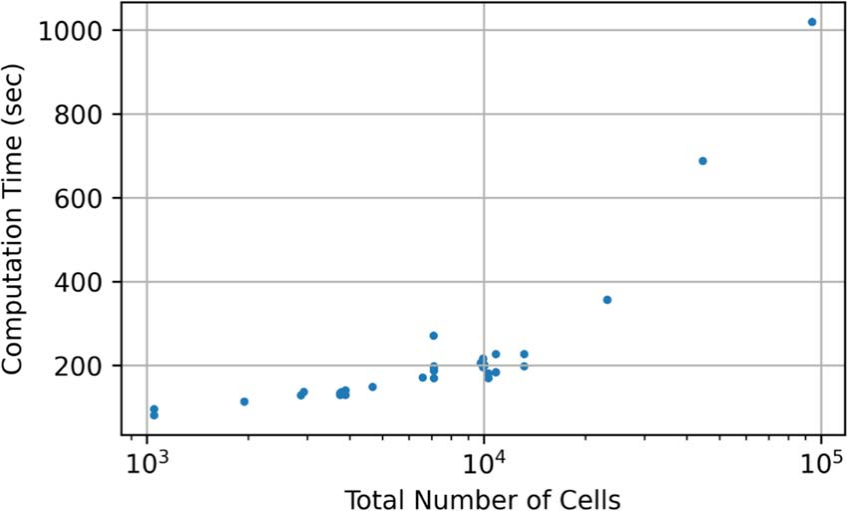
Run time of HiCat for annotating seen cell types experiment.

While HiCat provides a robust framework, its multi-step nature means computational cost is a consideration, particularly for very large datasets. HiCat’s design inherently manages some complexity; for instance, the significant dimensionality reduction to a concise 53-feature space makes the supervised learning step efficient. Users can further manage resources by leveraging the optimized implementations of HiCat’s established components (Harmony, UMAP, DBSCAN, CatBoost) and by tuning parameters within these tools to balance runtime with performance, alongside ensuring adequate hardware (CPU, memory). Figure 7 shows the scaling of run time with the total number of cell for HiCat runing on a 8-core 64GB RAM session.

### HiCat’s advantages in novel cell type discovery

A key strength of HiCat is its capacity to facilitate the discovery and differentiation of multiple unknown cell types, offering significant advantages over traditional, purely manual annotation workflows. While expert review remains invaluable for biological interpretation and final validation, HiCat automates the critical initial stages of novel cell type discovery. This data-driven, algorithmic framework enhances reproducibility and reduces the inherent subjectivity and potential expert bias often associated with manual exploration or gating strategies for identifying new cell types from scratch. Furthermore, HiCat’s automated pipeline provides a scalable solution for interrogating large and complex scRNA-seq datasets, where manually sifting for rare or unknown populations can be impractical. By integrating DBSCAN’s sensitivity to small, dense clusters with confidence scores from the supervised classifier, HiCat can improve the detection of potentially rare or subtly distinct novel cell types that might otherwise be overlooked. Crucially, this process directs expert attention toward a manageable set of well-delineated candidate novel populations, thereby streamlining subsequent characterization efforts, such as marker gene analysis and functional investigation, and fostering more efficient, data-driven hypothesis generation. It is important to distinguish this automated discovery role from the potential need for manual refinement in cases where, e.g. HiCat might delineate fine-grained subtypes of known cells as novel due to significant expression differences from the reference. For truly novel cell types initially identified by HiCat, subsequent expert review is then primarily focused on biological characterization and validation, rather than the *de novo* discovery which HiCat has already systematically facilitated.

### Limitations

HiCat performs well across various genomic data scenarios, yet certain limitations should be noted, highlighting opportunities for enhancement and exploration. Following are three examples.

Firstly, our method assumes that the reference and query sets contain enough cells in shared cell types, ensuring significant overlap between the two datasets. This facilitates effective alignment during batch effect removal since the Harmony algorithm utilizes shared cell types to align both reference and query data in a unified space. However, when the reference set holds cell types with numerous samples absent from the query set, Harmony’s performance may decline. Insufficient overlap can hinder its ability to align the datasets accurately, leading to suboptimal embeddings.

Secondly, each element of HiCat—like Harmony, UMAP, DBSCAN, and CatBoost—has been employed as standard methods with default settings in this research. Although this study aimed to showcase the pipeline’s overall capabilities in managing unseen cell types, tuning the parameters of each component could notably enhance performance. For instance, revising the number of UMAP neighbors or adjusting DBSCAN clustering parameters might yield more precise clustering and improved differentiation of novel cell types. Furthermore, optimizing the CatBoost model’s parameters, such as adjusting the learning rate or depth, could further boost classification accuracy. A major challenge in seeking optimal settings is the computational resources and datasets needed to thoroughly evaluate every possible parameter combination for each component within the HiCat pipeline. A comprehensive search for ideal configurations requires an extensive range of datasets across various conditions, which can be resource-intensive.

Thirdly, the primary aim of this paper is to present our methodology. The methods compared with HiCat in this work are selected based on their frequent use in comparisons with other published techniques, although newer and potentially better approaches exist. The 33 data pairs used in this study are designed to examine the cross-platform and cross-species performance of annotation methods, but here we have pooled all of them together for analysis.

Fourthly, the large-scale human lung case study revealed practical boundaries for HiCat’s novel discovery process. The analysis showed that when a novel subtype is transcriptionally very similar to an abundant population in the reference set, HiCat may ‘collapse’ the novel type into the known parent lineage (e.g. AT1 cells were classified as the known AT2 type). This suggests that HiCat’s discovery power is strongest for lineages that are clearly distinct from the reference populations. Additionally, the case study confirmed a practical lower limit for robust identification, as extremely rare populations ($n<20$) were sometimes absorbed into larger clusters or formed small, heterogeneous groups, reflecting a known challenge in clustering sparse data.

### Future directions

Building on the current HiCat framework, future work will focus on several key areas to address the method’s limitations and expand its capabilities. To improve performance in scenarios with limited reference-query overlap, we will investigate and integrate alternative batch effect correction methods, such as Seurat’s canonical correlation analysis or mutual nearest neighbors [69–71]. We will also move beyond the default settings used in this study by employing systematic hyperparameter optimization techniques, like grid search or Bayesian optimization [72], to pinpoint the most effective parameter combinations for all pipeline components. We will further explore the development of a more adaptive pipeline that can adjust settings dynamically based on data characteristics.

## Conclusion

HiCat presents a transformative solution for cell-type annotation by fusing the strengths of supervised and unsupervised learning, integrating genomic data from both reference and query sets, and utilizing a multi-resolution embedding space. Its adaptability, resilience, and capability to manage new cell types, even in limited populations, position it as a significant asset for genomic data analysis. Benchmark results convincingly show that HiCat surpasses current methods across multiple scenarios, especially when query datasets include new cell types, thereby highlighting its importance in intricate biological research.

**Key Points**

- Novel semi-supervised pipeline HiCat for annotating cell types from scRNA-seq data.
- Outperforms other supervised scRNA-seq cell type annotation methods.
- Discriminate between unseen cell types(cell types that are not present in the training set).

## Supplementary Material

datasetDetails_bbaf428

## Data Availability

All the scRNA-seq datasets used in this study are publicly available datasets, and their accession numbers could be found in datasetDetals.pdf. The code and scripts used for the analysis has been uploaded to GitHub: https://github.com/changbiHub/HiCat.

## References

[1] Jovic D, Liang X, Zeng H. et al. Single-cell RNA sequencing technologies and applications: a brief overview. *Clin Transl Med* 2022;12:e694. 10.1002/ctm2.694 35352511 PMC8964935

[2] Bridges K, Miller-Jensen K. Mapping and validation of scRNA-seq-derived cell-cell communication networks in the tumor microenvironment. *Front Immunol* 2022;13:885267. 10.3389/fimmu.2022.885267 35572582 PMC9096838

[3] Sallese MR, Riso PL, Villa CE. et al. Abstract A087: an ovarian cancer scRNA-seq atlas to dissect tumor-host interactions underlying metastatization and chemoresistance. *Cancer Res* 2024;84:A087. 10.1158/1538-7445.OVARIAN23-A087

[4] Zhang P, Liu J, Pei S. et al. Mast cell marker gene signature: prognosis and immunotherapy response prediction in lung adenocarcinoma through integrated scRNA-seq and bulk RNA-seq. *Front Immunol* 2023;14:1189520. 10.3389/fimmu.2023.1189520 37256127 PMC10225553

[5] Pasquini G, Arias JER, Schäfer P. et al. Automated methods for cell type annotation on scRNA-seq data. *Comput Struct Biotechnol J* 2021;19:961–9. 10.1016/j.csbj.2021.01.015 33613863 PMC7873570

[6] Liu Y, Li T, Wang Z. et al. Exploring parameter-efficient fine-tuning of a large-scale pre-trained model for scRNA-seq cell type annotation. In: Programs and Abstracts of the 2023 IEEE International Conference on Bioinformatics and Biomedicine (BIBM). pp. 580–5. Los Alamitos, CA: IEEE Computer Society, 2023.

[7] Stuart T, Butler A, Hoffman P. et al. Comprehensive integration of single-cell data. *Cell* 2019;177:1888–1902.e21. 10.1016/j.cell.2019.05.031 31178118 PMC6687398

[8] Pliner HA, Shendure J, Trapnell C. Supervised classification enables rapid annotation of cell atlases. *Nat Methods* 2019;16:983–6. 10.1038/s41592-019-0535-3 31501545 PMC6791524

[9] Lieberman Y, Rokach L, Shay T. CaSTLe—classification of single cells by transfer learning: harnessing the power of publicly available single cell RNA sequencing experiments to annotate new experiments. *PloS One* 2018;13:e0205499. 10.1371/journal.pone.0205499 30304022 PMC6179251

[10] Chen T, Guestrin C. XGBoost: a scalable tree boosting system. In: Proceedings of the 22nd ACM SIGKDD International Conference on Knowledge Discovery and Data Mining. New York, NY: Association for Computing Machinery (ACM), 2016, pp. 785–94.

[11] Tan Y, Cahan P. SingleCellNet: a computational tool to classify single cell RNA-Seq data across platforms and across species. *Cell Syst* 2019;9:207–213.e2. 10.1016/j.cels.2019.06.004 31377170 PMC6715530

[12] Breiman L . Random forests. *Mach Learn* 2001;45:5–32. 10.1023/A:1010933404324

[13] Alquicira-Hernandez J, Sathe A, Ji HP. et al. scPred: accurate supervised method for cell-type classification from single-cell RNA-seq data. *Genome Biol* 2019;20:264. 10.1186/s13059-019-1862-5 31829268 PMC6907144

[14] Wagner F, Yanai I. Moana: a robust and scalable cell type classification framework for single-cell RNA-Seq data. bioRxiv. 2018. 10.1101/456129

[15] Saunders C, Weston J, Stitson MO. et al. Support Vector Machine Reference Manual. Technical Report CSD-TR-98-03. Egham, UK: Department of Computer Science, Royal Holloway, University of London, 1998.

[16] Aran D, Looney AP, Liu L. et al. Reference-based analysis of lung single-cell sequencing reveals a transitional profibrotic macrophage. *Nat Immunol* 2019;20:163–72. 10.1038/s41590-018-0276-y 30643263 PMC6340744

[17] Kiselev VY, Yiu A, Hemberg M. scmap: projection of single-cell RNA-seq data across data sets. *Nat Methods* 2018;15:359–62. 10.1038/nmeth.4644 29608555

[18] de Kanter JK, Lijnzaad P, Candelli T. et al. CHETAH: a selective, hierarchical cell type identification method for single-cell RNA sequencing. *Nucleic Acids Res* 2019;47:e95. 10.1093/nar/gkz543 31226206 PMC6895264

[19] Hou R, Denisenko E, Forrest ARR. scMatch: a single-cell gene expression profile annotation tool using reference datasets. *Bioinformatics* 2019;35:4688–95. 10.1093/bioinformatics/btz292 31028376 PMC6853649

[20] Rui F, Gillen AE, Sheridan RM. et al. clustifyr: an R package for automated single-cell RNA sequencing cluster classification. *F1000Research* 2020;9:223. 32765839 10.12688/f1000research.22969.1 PMC7383722

[21] Atakan Ekiz H, Conley CJ, Zac Stephens W. et al. CIPR: a web-based R/shiny app and R package to annotate cell clusters in single cell RNA sequencing experiments. *BMC Bioinformatics* 2020;21:191. 10.1186/s12859-020-3538-2 32414321 PMC7227235

[22] Cover T, Hart P. Nearest neighbor pattern classification. *IEEE Trans Inf Theory* 1967;13:21–7. 10.1109/TIT.1967.1053964

[23] Wang S, Pisco AO, McGeever A. et al. Leveraging the cell ontology to classify unseen cell types. *Nat Commun* 2021;12:5556. 10.1038/s41467-021-25725-x 34548483 PMC8455606

[24] Chenling X, Lopez R, Mehlman E. et al. Probabilistic harmonization and annotation of single-cell transcriptomics data with deep generative models. *Mol Syst Biol* 2021;17:e9620. 10.15252/msb.20209620 33491336 PMC7829634

[25] Lin Y, Cao Y, Kim HJ. et al. scClassify: sample size estimation and multiscale classification of cells using single and multiple reference. *Mol Syst Biol* 2020;16:e9389. 10.15252/msb.20199389 32567229 PMC7306901

[26] Boufea K, Seth S, Batada NN. scID uses discriminant analysis to identify transcriptionally equivalent cell types across single-cell RNA-Seq data with batch effect. *iScience* 2020;23:100914. 10.1016/j.isci.2020.100914 32151972 PMC7063229

[27] Ji X, Tsao D, Bai K. et al. scAnnotate: an automated cell-type annotation tool for single-cell RNA-sequencing data. *Bioinform Adv* 2023;3:vbad030. 10.1093/bioadv/vbad030 36949780 PMC10027414

[28] Zhang Y, Zhang F, Wang Z. et al. scMAGIC: accurately annotating single cells using two rounds of reference-based classification. *Nucleic Acids Res* 2022;50:e43. 10.1093/nar/gkab1275 34986249 PMC9071478

[29] Xie P, Gao M, Wang C. et al. SuperCT: a supervised-learning framework for enhanced characterization of single-cell transcriptomic profiles. *Nucleic Acids Res* 2019;47:e48. 10.1093/nar/gkz116 30799483 PMC6486558

[30] Ma F, Pellegrini M. ACTINN: automated identification of cell types in single cell RNA sequencing. *Bioinformatics* 2020;36:533–8. 10.1093/bioinformatics/btz592 31359028

[31] Shao X, Yang H, Zhuang X. et al. scDeepSort: a pre-trained cell-type annotation method for single-cell transcriptomics using deep learning with a weighted graph neural network. *Nucleic Acids Res* 2021;49:e122. 10.1093/nar/gkab775 34500471 PMC8643674

[32] Chen X, Chen S, Song S. et al. Cell type annotation of single-cell chromatin accessibility data via supervised Bayesian embedding. *Nat Mach Intell* 2022;4:116–26. 10.1038/s42256-021-00432-w

[33] Wei Z, Zhang S. CALLR: a semi-supervised cell-type annotation method for single-cell RNA sequencing data. *Bioinformatics* 2021;37:i51–8. 10.1093/bioinformatics/btab286 34252936 PMC8686678

[34] Kimmel JC, Kelley DR. scNym: semi-supervised adversarial neural networks for single cell classification. *Genome Res* 2021;31:1781–93. 10.1101/gr.268581.120 33627475 PMC8494222

[35] Ganin Y, Ustinova E, Ajakan H. et al. Domain-Adversarial Training of Neural Networks. Journal of Machine Learning Research 2016;17:1–35.

[36] Zhongyuan X, Luo J, Xiong Z. scSemiGAN: a single-cell semi-supervised annotation and dimensionality reduction framework based on generative adversarial network. *Bioinformatics* 2022;38:5042–8. 36193998 10.1093/bioinformatics/btac652

[37] Goodfellow I, Pouget-Abadie J, Mirza M. et al. Generative adversarial nets. In: Ghahramani Z, Welling M, Cortes C, Lawrence ND, Weinberger KQ (eds), Advances in Neural Information Processing Systems, Vol. 27. pp. 2672–2680. Troy, NY: Curran Associates, Inc., 2014.

[38] Yang F, Wang W, Wang F. et al. scBERT as a large-scale pretrained deep language model for cell type annotation of single-cell RNA-seq data. *Nat Mach Intell* 2022;4:852–66. 10.1038/s42256-022-00534-z

[39] Korsunsky I, Millard N, Fan J. et al. Fast, sensitive and accurate integration of single-cell data with Harmony. *Nat Methods* 2019;16:1289–96. 10.1038/s41592-019-0619-0 31740819 PMC6884693

[40] Tran HTN, Ang KS, Chevrier M. et al. A benchmark of batch-effect correction methods for single-cell RNA sequencing data. *Genome Biol* 2020;21. 10.1186/s13059-019-1850-9 PMC6964114 31948481

[41] McInnes L, Healy J, Melville J. UMAP: Uniform Manifold Approximation and Projection for Dimension Reduction. Journal of Open Source Software 2018;3:861. 10.21105/joss.00861

[42] Ester M, Kriegel H-P, Sander J, Xu X. A density-based algorithm for discovering clusters in large spatial databases with noise. In: Proceedings of the Second International Conference on Knowledge Discovery and Data Mining; 1996; Portland, OR. pp. 226–231.

[43] Dorogush AV, Ershov V, Gulin A. CatBoost: gradient boosting with categorical features support. In: Advances in Neural Information Processing Systems, Vol. 31. pp. 5742–5752, 2018.

[44] Baron M, Veres A, Wolock SL. et al. A single-cell transcriptomic map of the human and mouse pancreas reveals inter- and intra-cell population structure. *Cell Syst* 2016;3:346–360.e4. 10.1016/j.cels.2016.08.011 27667365 PMC5228327

[45] Muraro MJ, Dharmadhikari G, Grün D. et al. A single-cell transcriptome atlas of the human pancreas. *Cell Syst* 2016;3:385–394.e3. 10.1016/j.cels.2016.09.002 27693023 PMC5092539

[46] Xin Y, Kim J, Okamoto H. et al. RNA sequencing of single human islet cells reveals type 2 diabetes genes. *Cell Metab* 2016;24:608–15. 10.1016/j.cmet.2016.08.018 27667665

[47] Wang YJ, Schug J, Won K-J. et al. Single-cell transcriptomics of the human endocrine pancreas. *Diabetes* 2016;65:3028–38. 10.2337/db16-0405 27364731 PMC5033269

[48] Segerstolpe A, Palasantza A, Eliasson P. et al. Single-cell transcriptome profiling of human pancreatic islets in health and type 2 diabetes. *Cell Metab* 2016;24:593–607. 10.1016/j.cmet.2016.08.020 27667667 PMC5069352

[49] Lawlor N, George J, Bolisetty M. et al. Single-cell transcriptomes identify human islet cell signatures and reveal cell-type-specific expression changes in type 2 diabetes. *Genome Res* 2017;27:208–22. 10.1101/gr.212720.116 27864352 PMC5287227

[50] Tabula Muris Consortium, Overall coordination, Logistical coordination, Organ collection and processing, Library preparation and sequencing, Computational data analysis, Cell type annotation, Writing group, Supplemental text writing group, and Principal investigators . Single-cell transcriptomics of 20 mouse organs creates a tabula Muris. *Nature* 2018;562:367–72. 10.1038/s41586-018-0590-4 30283141 PMC6642641

[51] Ding J, Adiconis X, Simmons SK. et al. Systematic comparison of single-cell and single-nucleus RNA-sequencing methods. *Nat Biotechnol* 2020;38:737–46. 10.1038/s41587-020-0465-8 32341560 PMC7289686

[52] Tasic B, Menon V, Nguyen TN. et al. Adult mouse cortical cell taxonomy revealed by single cell transcriptomics. *Nat Neurosci* 2016;19:335–46. 10.1038/nn.4216 26727548 PMC4985242

[53] Campbell JN, Macosko EZ, Fenselau H. et al. A molecular census of arcuate hypothalamus and median eminence cell types. *Nat Neurosci* 2017;20:484–96. 10.1038/nn.4495 28166221 PMC5323293

[54] Bai K, Xing L, Shao X. et al. PCLDA: A Cell Annotation Tool Using scRNA-Seq Data Based on Simple Statistics Methods. Computational and Structural Biotechnology Journal 2023;4:1–17. Amsterdam, Netherlands: Elsevier B.V. 10.1016/j.csbj.2025.07.019

[55] Hao Y, Stuart T, Kowalski MH. et al. Dictionary learning for integrative, multimodal and scalable single-cell analysis. *Nat Biotechnol* 2024;42:293–304. 10.1038/s41587-023-01767-y 37231261 PMC10928517

[56] Zhang Z, Luo D, Zhong X. et al. SCINA: a semi-supervised subtyping algorithm of single cells and bulk samples. *Genes* 2019;10:531. 10.3390/genes10070531 31336988 PMC6678337

[57] De Filippo K, Dudeck A, Hasenberg M. et al. Mast cell and macrophage chemokines CXCL1/CXCL2 control the early stage of neutrophil recruitment during tissue inflammation. *Blood* 2013;121:4930–7. 10.1182/blood-2013-02-486217 23645836

[58] Poto R, Marone G, Galli SJ. et al. Mast cells: a novel therapeutic avenue for cardiovascular diseases? *Cardiovasc Res* 2024;120:681–98. 10.1093/cvr/cvae066 38630620 PMC11135650

[59] Travaglini KJ, Nabhan AN, Penland L. et al. A molecular cell atlas of the human lung from single-cell RNA sequencing. *Nature* 2020;587:619–25. 10.1038/s41586-020-2922-4 33208946 PMC7704697

[60] Li M, Zhang X, Ang KS. et al. DISCO: a database of deeply integrated human single-cell omics data. *Nucleic Acids Res* 2022;50:D596–602. 10.1093/nar/gkab1020 34791375 PMC8728243

[61] Buettner F, Natarajan KN, Paolo Casale F. et al. Computational analysis of cell-to-cell heterogeneity in single-cell RNA-sequencing data reveals hidden subpopulations of cells. *Nat Biotechnol* 2015;33:155–60. 10.1038/nbt.3102 25599176

[62] King HW, Orban N, Riches JC. et al. Single-cell analysis of human B cell maturation predicts how antibody class switching shapes selection dynamics. Sci Immunol 2021;6:eabe6291. Washington, DC: American Association for the Advancement of Science. 10.1126/sciimmunol.abe6291 33579751

[63] Xiong Y-X, Zhang X-F. scDOT: enhancing single-cell RNA-Seq data annotation and uncovering novel cell types through multi-reference integration. *Brief Bioinform* 2024;25:bbae072. 10.1093/bib/bbae072 38436563 PMC10939303

[64] Duò A, Robinson MD, Soneson C. A systematic performance evaluation of clustering methods for single-cell RNA-seq data. *F1000Research* 2018;7:1141. 10.12688/f1000research.15666.2 30271584 PMC6134335

[65] Jiang J, Faiz A, Berg M. et al. Gene signatures from scRNA-seq accurately quantify mast cells in biopsies in asthma. *Clin Exp Allergy* 2020;50:1428–31. 10.1111/cea.13732 32935368 PMC7756890

[66] Yang Y, Jeong J, Tingting S. et al. Interleukin-7-based identification of liver lymphatic endothelial cells reveals their unique structural features. *JHEP Reports* 2024;6:101069. 10.1016/j.jhepr.2024.101069 38966234 PMC11222939

[67] LeBien TW, Tedder TF. B lymphocytes: how they develop and function. *Blood* 2008;112:1570–80. 10.1182/blood-2008-02-078071 18725575 PMC2518873

[68] Whitfield ML, George LK, Grant GD. et al. Common markers of proliferation. *Nat Rev Cancer* 2006;6:99–106. 10.1038/nrc1802 16491069

[69] Haghverdi L, Lun ATL, Morgan MD. et al. Batch effects in single-cell RNA-sequencing data are corrected by matching mutual nearest neighbors. *Nat Biotechnol* 2018;36:421–7. 10.1038/nbt.4091 29608177 PMC6152897

[70] Feng Zhang YW, Tian W. A novel approach to remove the batch effect of single-cell data. *Cell Discovery* 2019;5:1–4. 10.1038/s41421-019-0114-x 31636959 PMC6796914

[71] Yang Y, Li G, Qian H. et al. SMNN: batch effect correction for single-cell RNA-seq data via supervised mutual nearest neighbor detection. *Brief Bioinform* 2020;22:bbaa097. 10.1093/bib/bbaa097 PMC8324985 32591778

[72] Klein A, Falkner S, Bartels S. et al. Fast Bayesian hyperparameter optimization on large datasets. *Electron J Stat* 2017;11:4945–68.

