# Supplementary material for: HiCat: a semi-supervised approach for cell type annotation
Source: Brief Bioinform. 2025 Aug 20;26(4):bbaf428. doi: 10.1093/bib/bbaf428 (PMC12365967; doi:10.1093/bib/bbaf428)
Supplement: datasetDetails_bbaf428 [file datasetdetails_bbaf428.pdf]

	Reference							Query						
	Study	Organism and tissue	Sequence platform	No. of cells	No. of genes	No. of cell types	GEO or EBI number	Study	Organism and tissue	No. of cells	No. of genes	No. of cell types	Sequence platform	GEO or EBI number
**1**	Baron et al	Human pancreas	inDrop	8562	17500	13	GSE84133	Muraro et al	Human pancreas	2285	19054	13	CEL-seq2	GSE85241
**2**	Baron et al	Human pancreas	inDrop	8562	17500	13	GSE84133	Xin et al	Human pancreas	1492	33585	4	SMARTer	GSE81608
**3**	Muraro et al	Human pancreas	CEL-seq2	2285	19053	13	GSE85241	Baron et al	Human pancreas	8562	17500	13	inDrop	GSE84133
**4**	Muraro et al	Human pancreas	CEL-seq2	2285	19054	13	GSE85241	Xin et al	Human pancreas	1492	33585	4	SMARTer	GSE81608
**5**	Segerstolpe et al	Human pancreas	SMART-seq2	2394	22939	13	E-MTAB-5061	Muraro et al	Human pancreas	2285	19054	13	CEL-seq2	GSE85241
**6**	Campbell et al	Mouse hypothalamic arcuate-median eminence complex (HArc-ME)	Drop-seq	20921	26365	11	GSE93374	Tasic et al	Mouse primary visual cortex (PVC)	2285	19054	6	SMARTer	GSE71585
**7**	Ding et al.	Human PBMC	10x Chromium (v2)	3362	22757	9	GSE132044	Ding et al	Human PBMC	3222	21940	8	10x Chromium (v3)	GSE132044
**8**				3362	22757	9				6584	23528	9	Drop-seq	
**9**				3362	22757	9				6584	20033	9	inDrops	
**10**				3362	22757	9				3727	21591	7	Seq-Well	
**11**				3362	22757	9				526	22539	7	CEL-Seq2	
**12**				3362	22757	9				526	25374	7	Smart-seq2	
**13**			10x Chromium (v3)	3222	21940	8				526	22539	7	CEL-Seq2	
**14**				3222	21940	8				526	25374	7	Smart-seq2	
**15**			Drop-seq	6584	23529	9				3362	22757	9	10x Chromium (v2)	
**16**				6584	23529	9				3222	21940	8	10x Chromium (v3)	
**17**				6584	23529	9				6584	20033	9	inDrops	
**18**				6584	23529	9				3727	21591	7	Seq-Well	
**19**				6584	23529	9				526	22539	7	CEL-Seq2	
**20**				6584	23529	9				526	25374	7	Smart-seq2	
**21**			inDrops	6584	20033	9				3362	22757	9	10x Chromium (v2)	
**22**				6584	20033	9				3222	21940	8	10x Chromium (v3)	
**23**				6584	20033	9				6584	23529	9	Drop-seq	
**24**				6584	20033	9				3727	21591	7	Seq-Well	
**25**				6584	20033	9				526	22539	7	CEL-Seq2	
**26**				6584	20033	9				526	25374	7	Smart-seq2	
**27**			CEL-Seq2	526	22539	7				526	25374	7	Smart-seq2	
**28**			Smart-seq2	526	25374	7				526	22539	7	CEL-Seq2	
**29**	Schaum et al	Whole Mus musculus (TM）	SMART-Seq2	24622	22254	37	GSE109774	Schaum et al	Whole Mus musculus	20000	17867	32	10x	GSE109774
**30**	Schaum et al	Mus Lung	SMART-Seq2	1563	17,078	10	GSE109774	Schaum et al	Mus Lung	1303	13,718	8	10x	GSE109774
**31**	Muraro et al	Human pancreas	CEL-Seq2	2285	19053	13	GSE85241	Lawlor et al	Human pancreas	638	21563	13	Fluidigm C1	GSE86469
**32**	Zheng et al	PBMC	10X CHROMIUM	91,649	18,986	7	GSE132044	Zheng et al	PBMC	2467	13714	6	10x	GSE132044
**33**	Wang et al	Human pancreas	Human pancreas	457	19950	8	GSE83139	Xin et al	Human pancreas	1492	33585	4	SMARTer	GSE81608

Supplementary Table: dataset pair details. The row numbers are corresponding to the dataset index of the first two experiments.
